# Impacts of Microcystins on Morphological and Physiological Parameters of Agricultural Plants: A Review

**DOI:** 10.3390/plants10040639

**Published:** 2021-03-28

**Authors:** Alexandre Campos, El Mahdi Redouane, Marisa Freitas, Samuel Amaral, Tomé Azevedo, Leticia Loss, Csaba Máthé, Zakaria A. Mohamed, Brahim Oudra, Vitor Vasconcelos

**Affiliations:** 1 CIIMAR—Interdisciplinary Centre of Marine and Environmental Research, University of Porto, Terminal de Cruzeiros do Porto de Leixões, Av. General Norton de Matos, s/n, 4450-208 Porto, Portugal; maf@ess.ipp.pt (M.F.); samuel.amaral@ciimar.up.pt (S.A.); up200705237@fc.up.pt (T.A.); leticialoss@live.com (L.L.); vmvascon@fc.up.pt (V.V.); 2 Water, Biodiversity and Climate Change Laboratory, Phycology, Biotechnology and Environmental Toxicology Research Unit, Faculty of Sciences Semlalia Marrakech, Cadi Ayyad University, P.O. Box 2390, 40000 Marrakech, Morocco; redouane.elmahdii@gmail.com (E.M.R.); oudra@uca.ac.ma (B.O.); 3 ESS-P.Porto, School of Health, Polytechnic Institute of Porto, Rua Dr. António Bernardino de Almeida, 400, 4200-072 Porto, Portugal; 4 Department of Botany, Faculty of Science and Technology, University of Debrecen, 4032 Debrecen, Hungary; mathe.csaba@science.unideb.hu; 5 Department of Botany and Microbiology, Faculty of Science, Sohag University, Sohag 82524, Egypt; mzakaria_99@yahoo.com; 6 Department of Biology, Faculty of Sciences, University of Porto, Rua do Campo Alegre, 4069-007 Porto, Portugal

**Keywords:** harmful algal blooms, eutrophic waters, microcystins, agricultural plants, phytotoxicity, irrigation, agriculture, regulatory limits

## Abstract

Cyanobacteria are a group of photosynthetic prokaryotes that pose a great concern in the aquatic environments related to contamination and poisoning of wild life and humans. Some species of cyanobacteria produce potent toxins such as microcystins (MCs), which are extremely aggressive to several organisms, including animals and humans. In order to protect human health and prevent human exposure to this type of organisms and toxins, regulatory limits for MCs in drinking water have been established in most countries. In this regard, the World Health Organization (WHO) proposed 1 µg MCs/L as the highest acceptable concentration in drinking water. However, regulatory limits were not defined in waters used in other applications/activities, constituting a potential threat to the environment and to human health. Indeed, water contaminated with MCs or other cyanotoxins is recurrently used in agriculture and for crop and food production. Several deleterious effects of MCs including a decrease in growth, tissue necrosis, inhibition of photosynthesis and metabolic changes have been reported in plants leading to the impairment of crop productivity and economic loss. Studies have also revealed significant accumulation of MCs in edible tissues and plant organs, which raise concerns related to food safety. This work aims to systematize and analyze the information generated by previous scientific studies, namely on the phytotoxicity and the impact of MCs especially on growth, photosynthesis and productivity of agricultural plants. Morphological and physiological parameters of agronomic interest are overviewed in detail in this work, with the aim to evaluate the putative impact of MCs under field conditions. Finally, concentration-dependent effects are highlighted, as these can assist in future guidelines for irrigation waters and establish regulatory limits for MCs.

## 1. Introduction

Harmful algal blooms (HABs) constitute a real threat to aquatic ecosystems. In freshwater ecosystems, HABs are often composed by cyanobacteria and can be designated in this case as cyanobacterial harmful blooms (CHBs). The greatest danger associated with cyanobacteria is their ability to produce bioactive metabolites, some of which are toxic to many organisms, including plants, animals and humans [1,2]. On the other hand, cyanobacteria are organisms that adapt quite well to eutrophic environments and can, under favorable conditions, overcome the growth of other microalgae and form large green masses in the water column (blooms) [1,2]. The frequency and intensity of the occurrence of HABs is further enhanced by climate change and anthropogenic pollution (e.g., increase nutrient load in the aquatic environment) [3,4].

During an outbreak of toxic cyanobacteria, the toxins in the aquatic environment can reach alarming concentrations, i.e., exceeding the limit of 1 µg/L of microcystins (MCs) in drinking water proposed by the World Health Organization (WHO). The implications of the presence of cyanobacteria in the aquatic environment (especially freshwater environments) are related to the accumulation of toxins and their adverse effects, resulting in a decrease in aquatic biodiversity [2]. The use of low-quality water, with high concentrations of cyanotoxins, also constitute a threat to human health, with illnesses being associated to acute and chronic exposure to cyanotoxins. The most significant routes of exposure are the ingestion of contaminated water and food and dermal exposure [5].

The recurrent use of eutrophic waters containing high MC concentrations in agriculture is a matter of concern [6]. This practice can cause soil contamination, inhibition of plant growth and decrease in yield, changes in nutritional quality, as well as contamination of plant products. Indeed, the research carried out so far enabled us to identify some of the risks associated with the use of water contaminated with cyanotoxins in crop irrigation.

Microcystins are among all cyanotoxins, the group causing more damage to the environment and human health. These toxins are cyclic heptapeptides, containing in their composition a rare amino acid (2S, 3S, 8S, 9S) -3-amino-9-methoxy-2,6,8-trimethyl-10-phenyldeca-4,6 -dienoic acid (ADDA) [7]. The common structure of an MC is cyclo-(D-alanine-X-D-MeAsp-Z-Adda-D-glutamate-Mdha) in which X and Z are variable L amino acids [7]. When the variable amino acids leucine (L) and arginine (R) are present in positions X and Z, the molecule is designated microcystin-LR (MC-LR). Nevertheless, MCs are a very diverse chemical group with more than 200 chemical variants described [8]. The concerns expressed about this particular group of toxins are related to its increased toxicological potential, but also to its increased occurrence and persistence in the aquatic environment [9,10,11]. Indeed, MCs are found worldwide, in many different aquatic ecosystems and climates [9,10]. Moreover, MCs are found in about 40–75% of cyanobacterial blooms [12]. The cyanobacteria genera that produce MCs include *Microcystis*, *Anabaena*, *Nostoc*, *Oscillatoria*, *Anabaenopsis* and *Aphanocapsa* [7,8].

MCs are highly bioactive molecules. Their toxicity is generally associated to the interaction with protein phosphatases 1 and 2A and the inhibition of these enzymes [13,14,15]. Other molecular and cellular events characterizing the toxicity of MCs include reactive oxygen species (ROS) generation, oxidative stress, DNA damage, cell death and apoptosis [16,17].

**Figure 1:**
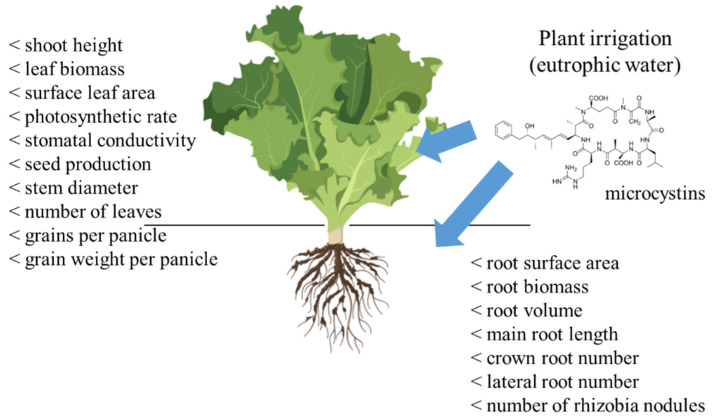
Main physiological and growth effects reported in agricultural plants irrigated or grown with microcystin (MC)-rich waters. Plant image is from BioRender (https://biorender.com/, accessed on 28 February 2021).

The presence of MCs in irrigation waters has been capturing the attention of the scientific community, since it represents a potential environmental and health problem. Studies have revealed that crop exposure to MCs, via irrigation, can effectively hinder their development and lead to crop contamination. MCs have shown to be particularly adverse in the early stages of plant development, i.e., during germination and seedling growth. Other adverse effects reported from exposure to MCs are the inhibition of plant growth, impairment of photosynthesis, tissue necrosis, oxidative stress, loss of membrane integrity and impairment in nutrient uptake [18,19,20,21]. Moreover, alterations mediated by MCs in cytoskeleton can affect mitotic processes and cause anomalies in tissue structure and plant development [22]. The main disturbances reported in the growth of agricultural plants are depicted in Figure 1.

In the research conducted so far, it stands out that not all plant exposure situations to MCs result in the impairment of plant growth and toxicity. In fact, the absence of adverse effects or even improved growth and performance have been observed. These responses are mostly related to exposure to low concentrations of toxins [23,24,25]. It is now evident that multiple factors contribute to the phytotoxicity of MCs. The results gathered so far demonstrate that there is a positive relation between plant injury or growth inhibition and toxin concentration in the irrigation water, as well as the length of the period of exposure. Nevertheless, the sensitivity of plants to MCs can vary considerably and according to the genotype, growth conditions and stage of development.

A meta-analysis of the research results published to date covering 35 crop plants [26] highlighted, for instance, that leafy vegetables such as dill, parsley and cabbage accumulate approximately three times more MCs in their edible tissues than other agricultural plants. An attempt was also made to relate the plant effects with the toxin concentration or dose of exposure. The analysis revealed that the impact of MCs in plants increase with the increase in exposure concentration. Changes in morphological parameters of 15–30% were linked to exposure to low-toxin doses (1 to 10 µg/L). This value increases up to 60% in plants exposed to low-medium toxin doses (10 to 100 µg/L) [26].

This work aims to systematize and analyze the most recent scientific achievements concerning the impacts of MCs in plant growth and physiology. Relevance will be given to research work covering realistic exposure scenarios and ecologically relevant MCs concentrations (e.g., investigations covering the effects of MCs in the range of 1–100 µg/L). Moreover, only works dedicated to agricultural species will be overviewed here. Finally, only morphological and physiological data related to plant growth and yield will be presented and discussed, as the primary focus of the present work is to evaluate the potential impact of MCs in plant productivity. Thereby, plant contamination data and related impacts will not be covered in detail in this work.

## 2. Plant Responses Related to Toxin Concentration and Chemical Structure

Small variations in the chemical structure of MCs were shown recently to have a significant impact on phytotoxicity. The variant (D-Leu1) MC-LR, found, for example, in La Plata Basin blooms in Argentina, proved to be more toxic than MC-LR to common bean (*Phaseolus vulgaris* L.) [27]. Malaissi et al. (2020) [27] reported the inhibition of germination after a single contact in the imbibition stage and delay in the development of common bean seedlings. Alterations in the structure of the leaves (alteration in the size, color and shape), roots (smaller root area) and stems (shorter stem lengths), as well as alterations in leaf stomatal density and conductivity were also reported (Table 1). A longer delay in the phototropic response was also reported. Some of these effects induced by (D-Leu1) MC-LR persisted over time, 30 days after a single contact with the toxin. Histological analysis showed significant alterations in the root tissues affecting the pericycle and endodermis and, for instance, an increase in the number of stomata on the abaxial side of *P. vulgaris* exposed to (D-Leu1) MC-LR [27]. Both MC variants induced lipid peroxidation and inhibited total phosphatase activity [27,28].

Liang and Liu (2020) [21] investigated the role of the phytohormones abscisic acid (ABA), indole-3-acetic acid (IAA), zeatin (ZT) and gibberellin (GA3) in the phytotoxicity of MCs. Plant hormones regulate plant development but are also important for other processes, for instance in the defense against stressors (Davies, 2010) [29]. The exposure of rice seedlings (*Oryza sativa* L.) to a diluted cyanobacterial extract with MCs (1, 10, 100 and 1000 µg MCs/L) revealed different types of responses in rice related to the concentration of the toxin. The growth and development of rice were promoted with the exposure to low concentrations of MCs (1 µg/L) (Table 1). This increase in growth coincided with the increase in the hormones IAA, ZT and GA3 in plant tissues. On the contrary, exposure to medium and high-toxin concentrations (10, 100 and 1000 µg MCs/L) inhibited rice growth (Table 1). This effect was accompanied by a decrease in IAA, ZT and GA3 and an increase in ABA. The changes in ABA levels were attributed to the increase in the expression of ABA biosynthetic genes OsNCED1, OsNCED3, OsNCED4 and OsZEP. The highest toxin concentration (1000 µg MCs/L) caused irreversible damage to the plants, preventing plants to recover growth after toxin exposure [21].

Other studies in rice have been revealing multiple morphological and physiological responses in this crop induced by MCs. Significant impairment in root development was reported in rice seedlings (7 days old) growing hydroponically [30,31]. One-month exposure to water contaminated with MCs (5, 50 and 500 µg/L) led to significant decrease in plant height, root dry weight, length, surface area and volume [30,31] (Table 1). Moreover, significant toxin accumulation in plants, particularly in the leaves, and alterations in the chemical composition of root exudates (organic acids, amino acids, sugars and dissolved organic carbon, DOC) were also reported [30]. Overall, impairment of growth was observed even in plants exposed to low-toxin concentration (5 µg/L) [30]. High bioavailability of MCs is expected in hydroponic cultures, and this can be reflected in an increased plant toxicity.

Toxin concentration effects were investigated in rice in three developmental stages [32]. The immediate effects of exposure of rice seedlings to increasing concentrations of MCs for 7 days consisted of the inhibition of seedling growth (reduced root, stem and leaf dry weight) and inhibition of photosynthesis (Table 1). The lowest toxin concentration causing growth inhibition was 100 µg/L. Moreover, some growth retardation symptoms induced by MCs (100 µg/L or higher toxin concentrations) persisted throughout the plant life cycle, denoting that seedling exposure, even for short periods, can be particularly critical and compromise subsequent stages of plant development. Mature plants displayed reduced numbers of grains per panicle, grain weight per panicle and setting percentage (Table 1). MCs were detected in all plant tissues and grains, in the plants exposed to 100 µg MCs/L or higher toxin concentrations [32]. Yet, short exposure (7 days) of rice seedlings and rice plants in the booting and filling stages to MCs (100–1000 µg/L) showed to affect grain production and quality. Among the alterations observed were a decrease in filled grains per panicle, a decrease in seed setting rate, panicle weight, soluble protein, sugar and starch in grain [33] (Table 1).

Other growth responses were reported in rice seedlings exposed to increasing MCs concentrations (1, 100, 1000 and 3000 µg MCs/L) [34]. In this study, the lowest toxin concentration (1 µg MCs/L) led to an increase in dry weight of roots, stems, leaves and plant height (Table 1). Nevertheless, 100 µg MCs/L or higher toxin concentrations significantly impaired the growth of rice seedlings (Table 1). The plants also accumulated considerable toxin amounts during exposure, the accumulation being positively correlated with the exposure concentrations. Seven days after exposure, the toxin accumulated in rice tissues decreased considerably [34].

Adult tomato (*Solanum lycopersicum* L.) plants (2 months old) showed reduced height, root length and leaf surface area, when exposed to MCs (3 and 6 µg/L) for 24 days [35] (Table 1). A decrease was also observed in chlorophyll and carbohydrate content in the plants due to irrigation with MC-rich water [35].

The effects of 10 and 50 µg MC-LR/L were also investigated in the root vegetable *Daucus carota* L. [25]. Plants grown for 28 days in soil were poorly affected with regard to growth and photosynthesis (measured by pulse amplitude modulation—PAM). The only adverse effect in growth was the decrease in root fresh weight in plants irrigated with water contaminated with 50 µg MC-LR/L (Table 1). Moreover, the authors reported changes in the content of minerals and vitamin C in carrot roots, highlighting that toxin exposure can affect the nutritional composition of this important root vegetable [25].

In lettuce (*Lactuca sativa* L.), exposure to MC-LR and MC-RR led to changes in photosynthesis related parameters, i.e., net-photosynthetic rate, transpiration and intercellular CO_2_ concentration (Pn) [36]. A gradual increase in all the parameters was reported, from day 1 to day 15, in most of toxin concentrations tested (Table 1). However, highest values were observed in plant groups exposed to low-toxin concentrations (0.65 and 2.5 µg MC-LR + MC-RR/L) (Table 1), denoting that low-toxin concentrations can promote photosynthesis. Low-toxin concentrations also increased stomatal conductance throughout the experiment. Surprisingly, the stimulation of photosynthesis did not affect plant growth. Nevertheless, the putative increase in photosynthesis could have been physiologically relevant for the plant, leading to the accumulation of metabolites, which could be essential in protecting cells against the toxic effects of MCs. Despite the absence of changes in growth, lettuce accumulated a considerable amount of MCs in the leaves [36].

Corbel et al. (2015) [37] carried out one of the longest studies of exposure with a fruit crop. Tomato plants were germinated and grown in soil irrigated daily with water contaminated with MCs (5.0 to 100.0 µg equivalent MC-LR/L) for 90 days. In this study, a remarkable observation was the absence of negative effects in growth and physiological parameters of tomato, even after plant exposure to 100.0 µg equivalent MC-LR/L. Of note was the anticipation of inflorescence and blooming stages in plants exposed to a low-toxin concentration (5.0 µg eq. MC-LR/L) (Table 1) [37].

Moreover, parsley (*Petroselinum crispum* L.) and coriander (*Coriandrum sativum* L.) plants (35 days old) growing in soil were unaffected by MCs [38] (Table 1). Plants irrigated for 10 days with water with 100, 500 and 1000 µg MCs/L, displayed biomass, chlorophyll and carotenoids and total protein values similar to control plants growing with clean (MC free) water. Moreover, there was no evidence in this study of an accumulation of MCs in parsley and coriander [38].

In addition to these findings, inhibition of *Zea mays* L., *Triticum aestivum* L., *Lepidium sativum* L. and *Medicago sativa* L. germination and growth were reported in earlier phytotoxicity studies carried out with pure toxin variants and cyanobacterial bloom material [39,40,41,42] (Table 1).

In summary, the studies reported above evidence two types of responses related to the concentration of MCs in irrigation water or culture medium. On the one hand, plant growth and physiological performance of plants can be stimulated, when plants are exposed to low concentrations of MCs [21,34,36]. However, most agricultural plants showed growth impairment when exposed to concentrations of MCs equal to or greater than 10 µg/L [21,25,32]. Growth impairment caused by MC concentrations below 10 µg/L were more frequent in the early stages of plant development (germination and seedling growth) [30]. Conversely, a study with tomato growing in soil revealed an absence of growth inhibition to MCs up to 100 µg/L [37]. In fact, the dualistic effects of MCs on plant growth could be related with some alterations in plants at the molecular level. On the one hand, there is the effect of the toxin in the phytohormones IAA, ZT and GA3, as mentioned above. The increase in the levels of these phytohormones by low concentrations of MCs will have a positive regulatory effect in plant metabolism and growth, whereas the opposite is verified with high-toxin concentrations [21]. On the other hand, low-toxin concentrations can lead to the activation of MAPK cascades [43]. This molecular activation in turn may affect several cellular metabolic processes regulated by MAPKs, resulting in a stimulation of plant metabolism and growth. MAPKs are, themselves, regulated by protein phosphatases, and their activation can result from a weak inhibition of protein phosphatases by MCs.

## 3. Plant Response Related with the Growth Conditions

Plants may manifest distinct physiological responses to MCs. As shown, morphological and physiological alterations are closely related with toxin concentrations. In soil-plant systems, however, the rhizo- and endosphere compartments play an important role in the manner plants respond to MCs. That is to say, MC bioavailability is highly influenced by the physicochemical properties of the soil and microbial activity of symbiotic microbiota. In this respect, Lahrouni et al. (2016) [56] investigated the phytotoxic effects of MCs on faba bean (*Vicia faba* L.) plants growing in soil and interacting with the symbiotic bacterial rhizobia. The results revealed that the exposure to MCs (100 µg/L), for 28 days, was detrimental to faba bean plants, decreasing root and shoot dry weight by 30–37 and 15%, respectively (Table 1). Similar inhibition results were observed in another study involving germination and hydroponic experiments [55]. Moreover, Lahrouni et al. (2016) [56] observed that MCs interfere with plant-rhizobia symbiosis, decreasing nodulation and nitrogen content in plants (Table 1). However, the authors observed that the phytotoxic effects of MCs were partially alleviated in plants inoculated with rhizobia. The protective effects were enhanced with the use of MC-tolerant rhizobia strains, which also retained significant nitrogen assimilation activity in plants [56]. On the same note, El Khalloufi et al. (2013) [51] reported the benefits of the use of MC-tolerant rhizobia strains, in the protection of *M. sativa* during MCs exposure. The symbiosis of this leguminous forage crop with MC-tolerant rhizobia diminished the stress symptoms in the plant, with plants showing less severe growth impairment. The use of rhizobial-mediated remediation to alleviate the adverse phytotoxic effects of MCs seems to be promising. Indeed, rhizobia were found to be capable of degrading MCs as previously reported by Zhu et al. (2016) [60]. Thereby, rhizobia may contribute to decreased toxin uptake and accumulation in plants. Zhu et al. (2016) [60] reported a novel isolate of *Rhizobium* sp., showing high MC-degrading activity, capable of hydrolyzing MC-LR from 8.3 mg/L to below the limits of detection within 10 h. Likewise, it was previously demonstrated that *Rhizobium gallicum* in consortium with *Microbacterium* sp. were able to degrade MCs at a faster rate (Ramani et al., 2012) [61]. Indeed, most literature suggests that the persistence of MCs in soil is mainly determined by the biotic degradation efficiency, especially the one carried out by microorganisms.

Soils with high content of organic matter seem to be propitious for the development of MCs-degrading microflora, and thereby to reduce the toxin load in soil and plant exposure to the toxin. The removal of the toxin within 7.1–17.8 days of half-life, on average, resulting in less MCs content in farm soils was reported (Chen et al., 2006) [62]. Other studies revealed that soil microbial activity increases the degradation of MCs (half-life of about 5 days) [63] by changing soil composition, i.e., enriching soil with humic acid and NaNO_3_. In contrast, MC degradation seemed to be significantly disrupted and inhibited when amending soil with NH_4_Cl, glycine and glucose or using high doses of phytosanitary products such as glyphosate and chlorothalonil.

On another note, Cao et al. (2017) [64] reported that high MC concentrations within the range of 100–1000 µg/L significantly decreased soil microbial metabolism related to phenol oxidation and carbon utilization potential. In addition, MCs reduced the abundance of ammonia-oxidizing bacteria and archaea in soil, resulting in less nitrification potential, and also may reduce the abundance of important plant growth-promoting rhizobacteria [64].

Furthermore, Xiang et al. (2020) [57] carried out a pot study to investigate the uptake and transfer of MCs in three leafy vegetables (*Ipomoea batatas* L., *Brassica juncea* L. and *Brassica alboglabra* L.). The soil-plant system was contaminated by adding pure toxin to the irrigation water (525 µg MC-LR/ L) or to the soil (150 µg MC-LR/ kg), or alternatively adding MC-producing cyano-bloom as manure (50 µg MC-LR / g). All three species were highly exposed to the phytotoxic impact of MCs (Table 1). Moreover, the toxin accumulation rate was significantly higher in the system contaminated with MC-producing cyano-bloom (intracellular MCs). One possible explanation for the differences in accumulation found is that the slow release of MCs from cyano-bloom material to the soil, after cellular breakdown mediated by soil microorganisms may have contributed to a continuous exposure and uptake of MCs in the roots, resulting in a high bioaccumulation rate. In contrast, free (extracellular) MCs added directly to the soil or in the irrigation water were rapidly degraded by the soil microflora, leading to a lower exposure and accumulation rate in plant tissues [54,62,65].

The above studies demonstrate that, in general, plants growing in soil-based systems are more protected than hydroponic cultures. This protective effect may be related with the bioavailability of the toxin that must be lower in soil-based cultures comparatively to hydroponic cultures. Indeed, in soil-based cultures, many processes will contribute to decrease MCs concentration, since the toxin may be (1) adsorbed onto clay minerals and organic matter, (2) flow to deeper soil layers [26,66,67] or (3) degraded [63]. In contrast, many of these processes do not occur in hydroponic cultures, and thereby higher concentrations of toxins are expected to be found in these systems, especially if MC-rich waters are used directly without previous treatment for elimination of contaminants. A recent meta-analysis investigation by Zhang et al. [26] revealed that MCs cause greater detrimental effects in plants growing in hydroponic systems than in soil. MCs inhibited photosynthetic efficiency by 57% in the hydroponic systems, but only by 31% in soil-based systems. Antioxidant enzyme activity was stimulated by 192% after exposure to MCs in the hydroponic systems, with approximately three times lower response (73% simulation) when plants were grown in soil. Nevertheless, according to our knowledge, there are no studies comparing the effects of MCs in plants growing in soil and hydroponic systems in a single experimental setup (Table 1). These kinds of studies are necessary, and the results must be taken in consideration for the future definition of guideline values of MCs concentration in waters used for agricultural purposes, since different limit concentrations can be set depending on the agricultural system.

Furthermore, we emphasize that the soil-plant systems have not been investigated in depth, demonstrating a clear lack of knowledge on how the whole system responds to the presence of MCs. It is, therefore, early to raise any conclusions about the safety and stability of soil-plant systems to MCs contamination. The true impact of MCs in soil microbial communities and how this will determine plant development require additional investigation. Finally, the use of a consortia of growth-promoting bacteria with the potential to degrade MCs seems to be a low-cost and environmentally relevant procedure that may protect agricultural plants from MC stress, besides appraising the underlying growth conditions in boosting this microbe-mediated biodegradation, whether in hydroponic or soil-based systems.

## 4. Plant Responses Related with the Stage of Development and Genotype

The relationship between the developmental stage and plant susceptibility to MCs was recently studied by Levizou et al. (2020) [47]. Radish (*Raphanus sativus* L.) and carrot seeds and seedlings in three stages of development, e.g., cotyledon, two-leaf and four-leaf stages, were exposed to raw water contaminated with MC-LR (2.03 μg/L) and MC-RR (1.43 μg/L) for 1.5 (radish) and 2.5 (carrot) months. The effects on growth were particularly noticeable in plants (radish and carrot) exposed to MCs during germination. Growth effects consisted in the decrease in the biomass of aerial part and taproot (Table 1). Inhibition of taproot growth also occurred in radish plants exposed during seedling stage (cotyledon and four leaves stages) to MCs rich water (Table 1). Additionally, the authors reported variable amounts of MCs accumulated in the taproots and in the leaves of the two root vegetables. These toxin quantities exceeded the WHO guideline value, making the produced taproots a health hazard to consumers [47]. Moreover, the authors also verified that the replacement of contaminated water and the subsequent irrigation of carrots with MC-free water for 2.5 months were not sufficient for plants to fully recover the normal growth, and MCs were still detected in carrot tissues [47]. Exposure to MCs also reduced the content of phenolic compounds in radish and carrots. Phenolic compounds play an important role in the defense against oxidative stress. In this respect, the decrease in phenolic content might render the plants more susceptible to the toxic effects of MCs [47]. Just like phenolic compounds, vitamins are also powerful antioxidant components in plants. This effect of MCs in the non-enzymatic antioxidant system of carrots are corroborated by the above-mentioned study from Machado et al. (2017) [25], in which MC-LR at 10 and 50 µg/L caused a decrease in vitamin C content in this vegetable root.

Species/genotype-related responses were revealed in a comparative investigation with rice and cucumber (*Cucumis sativus* L.) plants. The experiments carried out by Gu and Liang (2020) [49] showed that cucumber seedlings were more sensitive than rice to MCs. The differences in the sensitivity of the two plants were particularly marked after plant exposure to 5 μg MCs/L, the lowest toxin concentration tested. After 7 days of contact to 5 μg MCs/L, the relative growth rate (RGR) of cucumber seedlings decreased, whereas the RGR of rice seedlings increased (Table 1). The lowest toxin concentration affecting rice RGR was 10 μg/L (a decrease in RGR was observed after 5 days of exposure) (Table 1). Along with RGR inhibition, the authors observed an increase in ROS (O_2_^-^), H_2_O_2_ and lipid peroxidation, indicating oxidative stress in rice and cucumber. Concomitant to these results, the authors reported time- and toxin-concentration-specific variations in the activities of catalase (CAT), peroxidase (POD) and superoxide dismutase (SOD) and in gene expression, in both plant species [49].

Lettuce and rice seedlings were resistant to prolonged exposure to lake water contaminated with MCs [54]. The exposure to lake water with 2.61 and 5.22 μg MCs/L or to soil contaminated with 122.4 µg MCs /g soil, by mixing toxic cyanobacterial bloom material to the soil, did not affect growth and yield of both crops (Table 1). Growth parameters in lettuce such as shoot weight, root weight, leaf area, plant height, leaf area per tiller, numbers of productive ears and grain weight per tiller in rice were not affected during the entire exposure period to the toxin (Table 1). Despite the absence of alterations in growth, the authors reported significant accumulation of MCs in tissues of both crops, with the highest concentrations being observed in roots and in soil [54]. Similarly, a field study developed by Mohamed and Al-Shehri (2009) [68] reported an accumulation of MCs (10–1200 µg /Kg FW) in leaves and roots of six vegetable plants irrigated with groundwater containing 0.3–1.8 µg MCs/L, while the plants looked healthy.

Cucumber seedlings and plants in the early flowering and fruiting stages were investigated with regard to sensitivity to MCs [50]. Reduced plant height, stem diameter, number of leaves, root dry weight and shoot dry weight were observed in plants in all three growth stages exposed to the toxin for a period of 7 days (Table 1). However, cucumber seedlings demonstrated to be more sensitive to MCs, since they exhibited increased growth inhibition upon exposure to 10 µg MCs/L. In contrast, cucumber plants in the early flowering and fruiting stages lacked alterations in several growth parameters for the same treatment (exposure to 10 µg MCs/L) (Table 1). Growth inhibition was toxin-concentration-dependent. Moreover, toxin was accumulated in plant roots and fruits and high toxin accumulation coincided with the exposure to high MCs (100 and 1000 µg/L). Moreover, the impact of MCs in plant yield was striking. Cucumber plants exposed in the early stages of development to 10 µg MCs/L developed fruits with less weight and failed to produce fruits when exposed to high-toxin concentrations (100 and 1000 µg/L MCs). Exposure to MCs in the fruiting stage led plants to produce fruits with less weight and with a different nutritional composition. Plants exposed to 10 µg MCs/L, but not to 100 or 1000 µg MCs/L, were able to recover from the toxic effects of MCs, and growth parameters were similar to those of the control group after 7 days of growth without toxin [50].

Still, a long irrigation study (2 months) with raw water contaminated with MCs (5.11 µg/L) in lettuce revealed reduced sensitivity of this vegetable to the toxin [53]. Germinating seeds, but also seedlings in the cotyledon stage, or with two or four true leaves, were exposed to MCs. The only negative response of note was the decrease in root biomass and in the ratio root/shoot biomass. The decrease in root development was only observed in plants that received MCs during germination and young seedlings (cotyledon stage) (Table 1). Surprisingly, no changes were observed in the development of the aerial part of the plant and in photosynthesis parameters. Significant accumulation of MCs was found in leaves and roots of lettuce. The accumulation was higher in the plants exposed for a long period to the toxin, e.g., plants that received toxin from the germination stage to final harvest [53].

A soil study with lettuce, carrot and common bean plants revealed significant loss in yield parameters of these crops, upon exposure to MC-LR for 28 days. Toxin concentrations from 1 to 100 µg/L affected leaf length, total leaf mass and number of leaves in lettuce, as well as taproot mass and diameter in carrots and number of beans, bean length and bean mass in common bean [48] (Table 1).

Corbel et al. [45] analyzed the sensitivity of three crops, wheat, tomato (cultivars MicroTom and Saint-Pierre) and lettuce, to MCs. Germination tests revealed inhibition of wheat germination with 5 mg Eq MC-LR/L and higher concentrations (Table 1). The EC50 was 11 mg Eq MC-LR/L. Lower toxin concentrations (0.05 and 0.1 mg Eq MC-LR/L) increased the development of primary roots in the three crops, but higher concentrations (5-20 mg Eq MC-LR/L) were adverse and reduced root development in tomato and lettuce (Table 1). A soil experiment also carried out by the authors revealed a significant increase in the biomass of the aerial part of tomato plants exposed to 0.005 and 0.1 mg Eq MC-LR/L. The authors also reported disturbances in soil bacteria functioning and soil nitrification processes, which may also impact crop development [45].

An earlier study carried out by Pflugmacher et al. [44] revealed genotype-related responses in six spinach (*Spinacia oleracea* L.) cultivars exposed to MC-LR. Spinach plants were grown in semi-field conditions and irrigated with MC-LR (0.5 µg/L) for 6 weeks. Alterations in growth were detected only after 3 weeks of exposure. Spinach cultivars revealed variable levels of growth inhibition, leaf chlorosis and leaf size alterations. Photosynthetic oxygen production was also affected in all spinach cultivars (Table 1), meaning that photosynthesis was adversely affected with the exposure to MC-LR. The authors also reported alterations in antioxidative enzymes, indicating oxidative stress in spinach tissues [44].

Another species comparative study revealed that young rape (*Brassica napus* L.) plants are more sensitive than rice to MCs. Threshold concentrations causing a decrease in height in rice and rape were, respectively, 600 and 120 µg MCs/L [58] (Table 1).

From the above studies, it stands out that agricultural plants are more susceptible to MCs in early stages of development. For example, germination and seedling development were significantly affected by low and medium concentrations of MCs (e.g., concentrations up to 10 µg MCs/L) [47,50,54]. On the other hand, fewer adverse effects were reported in developed plants [50]. Exposure to toxins in the early stages of development, even for short periods, can significantly affect the subsequent stages of plant development and was shown to affect plant productivity [50]. The increased sensitivity of seedlings could be related to the differences in the metabolism at this stage of development and decreased intensity of defense responses. Evidence suggests that the antioxidant activity is lower in the first days of germination and increases during seedling development [69,70]. Finally, studies revealed that the effects of MCs are species-specific, showing increased MC-sensitivity of cucumber in relation to rice [49] and wheat in relation to tomato and lettuce, especially during germination [45]. Of note are the reports of absence of growth inhibition in lettuce and rice soil cultures exposed to MCs in concentrations close to 5 μg/L [53,54], in soil cultures of aromatic plants parsley and coriander exposed to 1000 μg MCs/L [38] and in soil cultures of tomato exposed to 100 μg/L MCs [45].

Finally, because the majority of the studies reported were carried out on leaf vegetables, further research is needed to assess the behavior of edible root and fruit vegetables, as these are also important human staple foods and highly cultivated worldwide.

## 5. Plant Responses Related to Combined Effects of MCs and Other Toxic Compounds and Stressors

In a recent study, radish seedlings growing in soil conditions were irrigated with tap water fortified with 2 and 12 µg MC-LR/L or with low-quality raw water (Karla reservoir, Greece) fortified with 12 µg/L MC-LR for 2 months [46]. Surprisingly, the authors reported very subtle (not significant) variations in radish growth (fresh weight) in virtually all experimental conditions studied. The only alteration of note was a decrease in the root fresh weight in plants exposed to Karla reservoir water fortified with MC-LR (Table 1). Moreover, radish photosynthetic rate and light use efficiency remained unaffected by the toxin and by the quality of the water used for irrigation. On the other hand, the authors reported a significant accumulation of MC-LR in roots (600–700 ng /g fw) and leaves (350–450 ng /g fw) of this vegetable. The effect produced in the soil microbiome by the eutrophic water from Karla reservoir is worth noting. However, the alterations observed were poorly related with the presence of the toxin in the water. This result reveals that other water quality parameters, besides MC-LR, may be detrimental to soil-plant systems [46].

Simple additive effects were reported in germinating lettuce seeds exposed to MC-LR and copper (Cu) mixture [52]. Moreover, synergistic and antagonistic effects on growth and antioxidative system of lettuce seedlings were observed for concentrations of MC-LR + Cu of, respectively, <50 + 500 µg/L and >1000 + 2000 µg/L. The authors reported also an increase in the accumulation of both contaminants, when present simultaneously in the plant growth medium. MC-LR alone did not affect plant development, and threshold concentrations causing decrease in plant fresh weight were 1000 µg Cu/L and 5 + 50 µg/L (MC-LR + Cu) (Table 1) [52].

The interactive effects of MCs and the toxic metal cadmium (Cd) were also investigated in rice [59]. Interestingly, the growth of rice in a hydroponic system inoculated with the toxic cyanobacterium *M. aeruginosa* (10^8^ cells/mL) for 3 weeks increased the growth of rice seedlings (increase root and shoot dry weight) and also increased total phosphorous (P) in the plant (Table 1). In fact, *M. aeruginosa* and Cd had antagonistic effects in rice, with the toxicity of Cd being partially alleviated by growing rice in a culture system inoculated with *M. aeruginosa*. Plants showed significant accumulation of both contaminants (Cd and MCs); however, the accumulation of one contaminant was inhibited by the presence of the other [59].

Freitas et al. (2015) [24] investigated the effects of mixtures of cyanotoxins MC-LR and cylindrospermopsin (CYN) in the growth and mineral uptake of lettuce. In this study, adult lettuce plants grown in nutrient solution (hydroponics) were exposed to environmental concentrations of MC-LR and CYN for 10 days. Lettuce demonstrated to tolerate a nutrient medium contaminated with MC-LR. Indeed, MC-LR stimulated plant growth at 1 and 10 µg/L, leading to an increase in root and leaf fresh weight (fw) after 10 days of exposure (Table 1). Surprisingly, plant growth was not affected by the exposure to 100 µg MC-LR/L. Nevertheless, the co-contamination of the culture system with 100 µg MC-LR + CYN/L was detrimental to the plant leading to a decrease in leaf biomass (Table 1). A significant alteration in mineral content was observed in lettuce leaves, indicating that both cyanotoxins interfere with mineral uptake and can affect the nutritional composition of this green vegetable. Moreover, the alterations in growth were variable and time- and toxin-concentration-dependent [24].

A recent study with spinach highlights precisely the synergistic effects of these two cyanotoxins [23]. Spinach hydroponic cultures exposed to MC-LR and CYN separately or combined for 21 days revealed for instance that 10 µg MC-LR/L or 10 µg CYN/L did not affect plant fresh weight or maximum fluorescence yield, thereby it may be considered not detrimental to the plant. Nevertheless, impairment of plant growth (decrease in leaf fresh weight) was observed when plants were exposed to the mixture of the two toxins in low doses (5 µg/L MC-LR + 5 µg/L CYN) (Table 1). The detrimental concentration of MC-LR and CYN when alone was 50 µg/L (Table 1) [23].

Moreover, it is important to note that most of these studies reporting effects of MCs in plants were carried out with raw water containing cyanobacterial blooms or cyanobacterial crude extracts. However, this cyanobacterial material contains many metabolites including other cyanotoxins, lipopolysaccharides and nutrients that can interfere with the assessment of MCs toxicity. Pereira et al. (2009) [71] reported the effects of aqueous extracts from *M. aeruginosa* strains (both microcystin-producing and non-producing strains) on germination and root growth of three important agricultural plant species: *Festuca rubra* L., *Lolium perenne* L. and *L. sativa L*. A clear inhibition of lettuce root growth exposed to strains containing MCs (5.9–56.4 μg/L) was found. Nevertheless, interestingly, the *M. aeruginosa* strain producing less MCs was the one causing most severe inhibition of root growth, which suggests that other metabolites present in *M. aeruginosa* extracts, besides MC-LR, may affect lettuce root growth. The studies performed by Pflugmacher et al. [40,42] (Table 1) revealed more pronounced impairments (germination and induction of oxidative stress) in *M. sativa* and *Z. mays* plants, upon exposure to crude extracts in comparison to pure toxin (e.g., MC-LR). The increased toxicity of natural cyano-bloom materials to plants (decreases of about 61% in growth) over cultured *M. aeruginosa* extracts or pure MCs was also highlighted in the meta-analysis investigation carried by Zhang et al. (2020) [26].

Overall, these works point to the possibility of synergistic interaction of MCs with other stress factors, leading to an increase in plant stress. In fact, the above works point to molecular synergies between MCs and other water pollutants such as metals (Cd and Cu) [52] and other types of cyanotoxins (CYN) [24]. We highlight, for example, the significant increase in the toxicity of MC-LR in lettuce, when present together with the metal Cu (5 MC-LR + 50 µg / L Cu) [52], and in lettuce and spinach, when present together with the cyanotoxin CYN [24]. Studies addressing the effects of the mixture of MCs with other emerging agricultural water/soil contaminants (e.g., pesticides) should be encouraged in the future, since the co-occurrence of different contaminants in the environment is highly predictable.

## 6. Conclusions

The research work carried out to date has revealed that the effects of MCs in agricultural plants are highly variable and strongly dependent on the concentration of MCs to which plants are exposed. However, the toxin concentration is not the only factor that determines the type of responses reported in plants and the extent of the phytotoxic damages. In this regard, we highlight that the time (duration) of exposure to the toxin, but also the plant genotype and stage of development, are factors to be considered when studying and evaluating the toxicity of MCs in plants. The highly variable responses reported in plants is making the assessment of regulatory limits for this toxin in irrigation waters a highly complex process. Plants are apparently more sensitive to MCs in the first stages of development (germination and seedling growth). Low and medium concentrations of toxin (up to 10 µg MCs/L) have been shown to inhibit germination and seedling development under different growth conditions in several plant species. On the other hand, well-developed plants were found to be relatively tolerant to this range of concentrations. Adverse effects on the growth of developed plants are associated with exposure to higher MCs concentrations (>10 µg/L). In addition, MC concentrations in the range of 1 to 5 µg/L were found to stimulate plant metabolism and growth.

Despite the results pointing to an increased sensitivity of plants in the early stages of development, the problem can be avoided if specific procedures in plant cultivation are followed. Indeed, germination and seedling development represent a short period in the plant life-cycle, requiring less consumption of water in comparison to the water needed for the subsequent development stages. Moreover, the initial stages of plant development often take place in nurseries, assisted by technology and with access to high-quality water. Thus, it is recommended to use high-quality (MC-free), instead of low-quality, water (with MCs), at least during plant germination and in the first stages of plan growth. The definition of a recovery period (irrigation with MC-free water) after a period of exposure to the toxin can also contribute to mitigate the effects of MCs, since studies have shown that the growth of some agricultural plants (e.g., rice) can partially recover growth and eliminate the toxin, following a period of growth in the absence of MCs.

The majority of the agricultural plants were found to tolerate MC concentrations up to 10 µg/L, after overcoming the initial seedling stage. Taking this into account, waters contaminated with MCs to maximum 10 µg/L will have a minor impact on plant growth, yield and quality, and thereby may be considered to define, in the future, a regulatory limit for irrigation waters.

It also seems that plants are more severely affected by the toxin when growing in hydroponic cultivation systems in comparison to soil cultures. This particular plant condition can be related with an increase in the bioavailability of the toxin in the hydroponic cultures compared with soil cultures. This does not mean that soil cultures are safer and preferred to hydroponic cultures. With regard to soil cultures, we underline the need of more investigation given the complexity of this system. Of the utmost relevance is the identification of the factors affecting the bioavailability of MCs in soil and the toxicity of MCs to soil organisms and to the overall biological activity of the soil.

Finally, along with plant development and productivity impairments, MC-rich irrigation water may also lead to the contamination of food produced by crops and human exposure to MCs via consumption of contaminated food products. These concerns require adequate analysis in the future and may help to clarify the safety limit that can be proposed for irrigation waters.

The contamination of surface waters with HABs is a complex process involving many factors and is difficult to control. The best way to act is preventing water eutrophication and to reduce nutrient loading in the aquatic environments. Green technologies such as constructed wetlands, on the other hand, can be extremely useful for recovering waters contaminated with HABs in a cost-effective way. Eventually, these technologies can be applied to provide better quality water to agriculture.

## Figures and Tables

**Table 1:** Overview of morphological and physiological effects in plants related to MCs concentrations reported in the literature. The information is organized by species and study. Moreover, plant species were ordered on the basis of species sensitivity to MCs, taking into account that this assessment depends on the investigation performed (germination test, hydroponics, soil experiment). No changes observed (nd); relative growth rate (RGR); pure toxin (*); crude extract or natural lake/reservoir water containing MCs (#); information not available (--); irrigation with polluted water (IPW); cultivation with polluted soil (CPS); application of cyanobacterial manure (ACM); 2, 4, 4′-Trichlorobiphenyl (PCB-28).

Plant Species	Growth Stage ^(1)^	Type Experiment/Exposure Time (days) ^(2)^	Toxin ^(3)^	Toxin Conc. (µg/L) ^(4)^	Physiological and Morphological Parameters ^(5)^	Reference
*EUDICOTS*						
*Spinacia oleracea (6 cultivars)*	Seedlings	Soil culture/42	MC-LR #	0.5	< photosynthetic oxygen production	[44]
*Spinacia oleracea*	Developed plants	Hydroponics/21	MC-LR #	50	< leaf fresh weight	[23]
			MC-LR, CYN #	5 and 25		
*Lepidium sativum*	Seeds	Germination/6	MC-LR #*	10	< total fresh weight	[41]
				1–10	< root length; leaf length	
*Solanum lycopersicum*	Seeds	Germination/7	MCs	50 and 100	> radicle length	[45]
				500–20,000	< radicle length	
		Soil culture/14		5–100	> shoot dry weight	
	Seeds	Soil culture/90	MCs	5	Anticipation of bloom of first flower; anticipation of first inflorescence emergence	[37]
	Developed plants	Soil culture/24	MCs	3–6	< inhibition of root length; carbohydrate content	[35]
				6	< chlorophyll content; inhibition of stem length; decrease in surface leaf area	
*Raphanus sativus*	Seedlings	Soil culture/64	MC-LR *#	raw water + MC-LR	< taproot fresh weight	[46]
	1-seeds	Soil culture 45	MC-LR + MC-RR #	3.76	< leaf biomass; taproot biomass; taproot volume	[47]
	2-seedlings				< taproot biomass; taproot volume	
*Daucus carota*	Seeds	Soil culture/75	MC-LR + MC-RR #	3.76	< leaf biomass; taproot biomass; taproot volume	[47]
	Developed plants	Soil culture/28	MC-LR *	1–100	< taproot mass and taproot diameter; taproot diameter	[48]
	Developed plants	Soil experiment/32	MC-LR #	50	< root fresh weight; < ascorbic acid (vitamin c)	[25]
				10	> max. fluorescence yield	
*Cucumis sativus*	Seedlings	Hydroponics/7	MCs	5	< RGR; > H2O2, O2^−^, MDA	[49]
	1-seedlings	Hydroponics/7	MCs	1	< plant height	[50]
				10–1000	< plant height; stem diameter; number of leaves; leaf area; root dry weight; shoot dry weight; yield	
	2-flowering				< plant height; stem diameter; leaf area	
				100–1000	< number of leaves; shoot dry weight; root dry weight; yield	
	3-fruiting			10–1000	< plant height; leaf area; root dry weight	
				100–1000	< stem diameter; number of leaves; shoot dry weight; yield	
*Medicago sativa*	Seeds	Germination/7	MC-LR, MC-LW *#	5	< length of primary root; > lipid peroxidation	[42]
	Seedlings	Irrigation/30	MCs #	5–20	< shoot dry weight; root dry weight; nodules dry weight	[51]
				10–20	< root nodule number	
*Lactuca sativa*	1-seeds	Germination/3	MC-LR *, Cu	50	< germination	[52]
	2-seedlings	Hydroponics/14		5	< total fresh weight	
				50	< root length	
				1000	< shoot length	
	1-seeds	Soil experiment/60	MCs	5.11	< root biomass; root/shoot biomass; > chlorophyll content	[53]
	2-seedling -cotyledon stage				< root biomass; root/shoot biomass	
	3-seedling with 2 and 4 leaves				nc	
	Seeds	Germination/7		50–5000	> radicle length	[45]
	Seedlings	Soil culture/60		2.61, 5.22	nc	[54]
	Developed plants	Soil culture/15	MC-LR + MC-RR #	0.65–13	> net photosynthetic rate; leaf tissue transpiration; intercellular CO_2_ concentration	[36]
				0.65 and 2.5	> stomatal conductance	
	Developed plants	Hydroponics/10	MC-LR *	1 and 10	> leaf biomass	[24]
				1 and 100	> root biomass	
			MC-LR + CYN *	100	< leaf biomass	
	Developed plants	Soil culture/28	MC-LR *	5–100	< leaf length	[48]
				1–100	< total leaf mass; number of leaves	
*Vicia faba*	1-seeds	Germination/7	MCs	50 and 100	< germination	[55]
	2-seedlings	Hydroponics/48			< shoot dry weight; root dry weight; rhizobia nodules dry weight	
	2-seedlings				< shoot and root dry weight; rhizobia nodules dry weight	
				10–100	< number of rhizobia nodules	
	Seedlings	Soil culture/28	MCs	100	< shoot and root dry weight; root nodule number	[56]
*Ipomoea batatas*	Developed plants	Soil culture/10	MC-LR #*, ACM	150	< main root length	[57]
			MC-LR #*, IPW, CPS, ACM		< total weight; aerial part weight	
*Brassica juncea*	Developed plants	Soil culture/10	MC-LR #*, IPW, ACM	150	< plant height; total weight	[57]
			MC-LR #*, IPW, CPS, ACM		< main root length; aerial part weight	
*Brassica alboglabra*	Developed plants	Soil culture/10	MC-LR #*, IPW	150	< main root length	[57]
			MC-LR #*, IPW, CPS, ACM		< plant height; total weight; aerial part weight	
*Petroselinum crispum*	Developed plants	Soil culture/7	MCs	100–1000	nc	[38]
*Coriandrum sativum*	Developed plants	Soil culture/7	MCs	100–1000	nc	[38]
*Brassica napus*	Seeds	Germination/10d	MC-RR, MC-LR, MC-YR #	600–3000	< germination	[58]
				120–3000	< plant height	
*Phaseolus vulgaris*	Seeds	Germination/1	(D-Leu1) MC-LR *	3500	< germination; > stomatal density and conductivity	[27]
			(D-Leu1) MC-LR and MC-LR *		delay seedling development; > morphological anomalies; delay phototropic response	
	Seeds	Germination/1	(D-Leu1) MC-LR and MC-LR *	3500	< chlorophyll content; delay seedling development	[28]
				15000	> lipid peroxidation	
			(D-Leu1) MC-LRR *	3500	< germination	
	Developed plants	Soil culture/28	MC-LR *	1 and 5	> bean length	[48]
				10-100	< bean mass; number of beans	
***MONOCOTS***						
*Triticum aestivum*	1-seeds	Germination/3	MC-RR, MC-LR #	0.5	< germination	[40]
	2-seedlings	Soil culture/15			< shoot length; root length; photosynthetic oxygen production	
*Triticum aestivum*	Seeds	Germination/7	MCs	5000–20000	< germination	[45]
				50 and 100	> radicle length	
				20000	< radicle length	
*Zea mays*	Seeds	Germination/--	MCs (6 analogues) #*	5	< germination; shoot length; root length	[39]
*Oryza sativa*	Seeds	Germination/10	MC-RR, MC-LR, MC-YR #	600–3000	< plant height	[58]
				120–3000	< root length; root fresh weight	
	Seedlings	Soil culture/60	MCs	2.61, 5.22	nc	[54]
	Seedlings	Hydroponics/30	MCs	5–500	< root dry weight	[30]
				50–500	< root length, surface area and volume; root surface area; root volume; lateral root number	
				500	< crown root number	
	Seedlings	Hydroponics/30	MCs	50–500	< plant height; root length; shoot dry weight; >membrane permeability	[31]
				5–500	< root dry weight	
	Seedlings	Hydroponics/7	MCs	1	> root biomass	[32]
				1–3000	< stem biomass	
				100–3000	< leaves biomass	
				1000–3000	< root biomass; grains per panicle; grain weight per panicle; setting percentage	
	Seedlings	Hydroponics/7	MCs	1	> root surface area, shoot height	[21]
				10	< root surface area	
				100	< shoot height	
	Seedlings	Hydroponics/7	MCs	1	> leaves dry weight; stem dry weight; roots dry weight	[34]
				100–3000	< leaves dry weight; stem dry weight; roots dry weight; net photosynthetic rate (Pn)	
	Seedlings	Hydroponics/7	MCs	5	> H_2_O_2_, O_2-_, MDA	[49]
				10	< RGR	
	Seedlings	Hydroponics/21	MCs	10	> shoot and root dry weight; P content in shoots and roots	[59]
			MCs, Cd	10	< root dry weight	
	1-seedlings	Hydroponics/7	MCs	10–1000	< root surface area	[33]
				100–1000	< plant height; Net assimilation rate; filled grains per panicle; seed setting rate; panicle weight; soluble protein, sugar and starch in grain	
	2-booting			100–1000	< plant height; root surface area; Net assimilation rate; filled grains per panicle; seed setting rate; panicle weight; soluble protein, sugar and starch in grain	
	3-filling			10–1000	< seed setting rate	
				100–1000	< root surface area; Net assimilation rate; filled grains per panicle; panicle weight; soluble protein, sugar and starch in grain	
				1000	< plant height	

^(1).^ The numbers in this column indicate the different experiments performed in the study. ^(2).^ Experiments were categorized as follows: germination, hydroponics and soil cultures. The time plants were exposed to the toxin is expressed in days. ^(3).^ Unless otherwise stated, the source of toxin is natural or cultured cyanobacterial bloom material. Microcystin variants reported are those identified and quantified by the authors. The abbreviation “MCs” is used when no information is available concerning the chemical variants present in toxic material. Other contaminants, or specific treatment conditions investigated in combination with the toxin, are also indicated. ^(4).^ Concentration of MCs causing the effect ^(5).^ Main morphological and physiological parameters measured in the study.

## Data Availability

Not applicable.

## References

[1] Buratti F.M., Manganelli M., Vichi S., Stefanelli M., Scardala S., Testai E., Funari E. (2017). Cyanotoxins: Producing organisms, occurrence, toxicity, mechanism of action and human health toxicological risk evaluation. Arch. Toxicol..

[2] Svirčev Z.B., Tokodi N., Drobac D., Codd G.A. (2014). Cyanobacteria in aquatic ecosystems in Serbia: Effects on water quality, human health and biodiversity. Syst. Biodivers..

[3] Paerl H.W., Huisman J. (2009). Climate change: A catalyst for global expansion of harmful cyanobacterial blooms. Environ. Microbiol. Rep..

[4] Gobler C.J. (2020). Climate Change and Harmful Algal Blooms: Insights and perspective. Harmful Algae.

[5] Cordeiro-Araújo M.K., Chia M.A., do Carmo Bittencourt-Oliveira M. (2017). Potential human health risk assessment of cylindrospermopsin accumulation and depuration in lettuce and arugula. Harmful Algae.

[6] Saqrane S., Oudra B. (2009). CyanoHAB occurrence and water irrigation cyanotoxin contamination: Ecological impacts and potential health risks. Toxins.

[7] Van Apeldoorn M.E., Van Egmond H.P., Speijers G.J.A., Bakker G.J.I. (2007). Toxins of cyanobacteria. Mol. Nutr. Food Res..

[8] Catherine A., Bernard C., Spoof L., Bruno M., Meriluoto J., Lisa Spoof L., Codd G.A. (2017). Microcystins and Nodularins. Handbook of Cyanobacterial Monitoring and Cyanotoxin Analysis.

[9] Díez-Quijada L., Prieto A.I., Guzmán-Guillén R., Jos A., Cameán A.M. (2019). Occurrence and toxicity of microcystin congeners other than MC-LR and MC-RR: A review. Food Chem. Toxicol..

[10] Graham J.L., Loftin K.A., Meyer M.T., Ziegler A.C. (2010). Cyanotoxin mixtures and taste-and-odor compounds in cyanobacterial blooms from the midwestern united states. Environ. Sci. Technol..

[11] Corbel S., Mougin C., Bouaïcha N. (2014). Cyanobacterial toxins: Modes of actions, fate in aquatic and soil ecosystems, phytotoxicity and bioaccumulation in agricultural crops. Chemosphere.

[12] Sivonen K., Jones G., Chorus I., Bartram J. (1999). Cyanobacterial Toxins. Toxic Cyanobacteria in Water: A guide to their public health consequences, monitoring and management.

[13] Craig M., Luu H.A., McCready T.L., Williams D., Andersen R.J., Holmes C.F.B. (1996). Molecular mechanisms underlying the interaction of motuporin and microycystins with type-1 and type-2A protein phosphatases. Biochem. Cell Biol. Biol. Cell..

[14] Liang J., Li T., Zhang Y.-L., Guo Z.-L., Xu L.-H. (2011). Effect of microcystin-LR on protein phosphatase 2A and its function in human amniotic epithelial cells. J. Zhejiang Univ. Sci. B.

[15] Christen V., Meili N., Fent K. (2013). Microcystin-LR induces endoplasmatic reticulum stress and leads to induction of NFκB, interferon-alpha, and tumor necrosis factor-alpha. Environ. Sci. Technol..

[16] Valério E., Vasconcelos V., Campos A. (2016). New Insights on the Mode of Action of Microcystins in Animal Cells—A Review. Mini-Rev. Med. Chem..

[17] IARC (2010). Ingested Nitrate and Nitrite, and Cyanobacterial Peptide Toxins.

[18] Abe T., Lawson T., Weyers J.D.B., Codd G.A. (1996). Microcystin-LR inhibits photosynthesis of *Phaseolus vulgaris* primary leaves: Implications for current spray irrigation practice. New Phytol..

[19] Kurki-Helasmo K., Meriluoto J. (1998). Microcystin uptake inhibits growth and protein phosphatase activity in mustard (*Sinapis alba* L.) seedlings. Toxicon.

[20] McElhiney J., Lawton L.A., Leifert C. (2001). Investigations into the inhibitory effects of microcystins on plant growth, and the toxicity of plant tissues following exposure. Toxicon.

[21] Liang C., Liu H. (2020). Response of hormone in rice seedlings to irrigation contaminated with cyanobacterial extract containing microcystins. Chemosphere.

[22] Garda T., Kónya Z., Tándor I., Beyer D., Vasas G., Erdodi F., Vereb G., Papp G., Riba M., M-Hamvas M. (2016). Microcystin-LR induces mitotic spindle assembly disorders in *Vicia faba* by protein phosphatase inhibition and not reactive oxygen species induction. J. Plant. Physiol..

[23] Llana-Ruiz-Cabello M., Jos A., Cameán A., Oliveira F., Barreiro A., Machado J., Azevedo J., Pinto E., Almeida A., Campos A. (2019). Analysis of the Use of Cylindrospermopsin and/or Microcystin-Contaminated Water in the Growth, Mineral Content, and Contamination of Spinacia oleracea and Lactuca sativa. Toxins.

[24] Freitas M., Azevedo J., Pinto E., Neves J., Campos A., Vasconcelos V. (2015). Effects of microcystin-LR, cylindrospermopsin and a microcystin-LR/cylindrospermopsin mixture on growth, oxidative stress and mineral content in lettuce plants (*Lactuca sativa* L.). Ecotoxicol. Environ. Saf..

[25] Machado J., Azevedo J., Freitas M., Pinto E., Almeida A., Vasconcelos V., Campos A. (2017). Analysis of the use of microcystin-contaminated water in the growth and nutritional quality of the root-vegetable, Daucus carota. Environ. Sci. Pollut. Res..

[26] Zhang Y., Whalen J.K., Sauvé S. (2020). Phytotoxicity and bioconcentration of microcystins in agricultural plants: Meta-analysis and risk assessment. Environ. Pollut..

[27] Malaissi L., Vaccarini C.A., Hernández M.P., Ruscitti M., Arango C., Busquets F., Arambarri A.M., Giannuzzi L., Andrinolo D., Sedan D. (2020). [D-Leu1]MC-LR and MC-LR: A Small–Large Difference: Significantly Different Effects on *Phaseolus vulgaris* L. (Fabaceae) Growth and Phototropic Response after Single Contact during Imbibition with Each of These Microcystin Variants. Toxins.

[28] Sedan D., Malaissi L., Vaccarini C.A., Ventosi E., Laguens M., Rosso L., Giannuzzi L., Andrinolo D. (2020). [D-Leu1]MC-LR Has Lower PP1 Inhibitory Capability and Greater Toxic Potency than MC-LR in Animal and Plant Tissues. Toxins.

[29] Davies P.J., Davies P.J. (2010). The plant hormones: Their nature, occurrence, and functions. Plant Hormones.

[30] Cao Q., Rediske R.R., Yao L., Xie L. (2018). Effect of microcystins on root growth, oxidative response, and exudation of rice (*Oryza sativa*). Ecotoxicol. Environ. Saf..

[31] Cao Q., Steinman A.D., Yao L., Xie L. (2017). Increment of root membrane permeability caused by microcystins result in more elements uptake in rice (*Oryza sativa*). Ecotoxicol. Environ. Saf..

[32] Liang C., Wang W., Wang Y. (2016). Effect of irrigation with microcystins-contaminated water on growth, yield and grain quality of rice (*Oryza sativa*). Environ. Earth Sci..

[33] Liang C., Ma X., Liu H. (2020). Effect of microcystins at different rice growth stages on its yield, quality, and safety. Environ. Sci. Pollut. Res..

[34] Liang C., Wang W. (2015). Response and recovery of rice (*Oryza sativa*) seedlings to irrigation with microcystin-contaminated water. Environ. Earth Sci..

[35] Al-Sultan E., Yousif A., Hatem M.T. (2019). Toxic effects of purified microcystins from soil blue-green alga oscillatoria pseudogeminata on tomato plant lycopersicon esculentum. Baghdad Sci. J..

[36] do Carmo Bittencourt-Oliveira M., Cordeiro-Araújo M.K., Chia M.A., de Toledo Arruda-Neto J.D., de Oliveira Ê.T., dos Santos F. (2016). Lettuce irrigated with contaminated water: Photosynthetic effects, antioxidative response and bioaccumulation of microcystin congeners. Ecotoxicol. Environ. Saf..

[37] Corbel S., Bouaïcha N., Nélieu S., Mougin C. (2015). Soil irrigation with water and toxic cyanobacterial microcystins accelerates tomato development. Environ. Chem. Lett..

[38] Pereira A.L., Azevedo J., Vasconcelos V. (2017). Assessment of uptake and phytotoxicity of cyanobacterial extracts containing microcystins or cylindrospermopsin on parsley (*Petroselinum crispum* L.) and coriander (*Coriandrum sativum* L.). Environ. Sci. Pollut. Res..

[39] Pflugmacher S. (2007). Reduction in germination rate and elevation of peroxidase activity in Zea mays seedlings due to exposure to different microcystin analogues and toxic cell free cyanobacterial crude extract. J. Appl. Bot. Food Qual..

[40] Pflugmacher S., Hofmann J., Hübner B. (2007). Effects on growth and physiological parameters in wheat (*Triticum aestivum* L.) grown in soil and irrigated with cyanobacterial toxin contaminated water. Environ. Toxicol. Chem..

[41] Gehringer M.M., Kewada V., Coates N., Downing T.G. (2003). The use of Lepidium sativum in a plant bioassay system for the detection of microcystin-LR. Toxicon.

[42] Pflugmacher S., Jung K., Lundvall L., Neumann S., Peuthert A. (2006). Effects of cyanobacterial toxins and cyanobacterial cell-free crude extract on germination of alfalfa (*Medicago sativa*) and induction of oxidative stress. Environ. Toxicol. Chem..

[43] Gehringer M.M. (2004). Microcystin-LR and okadaic acid-induced cellular effects: A dualistic response. FEBS Lett..

[44] Pflugmacher S., Aulhorn M., Grimm B. (2007). Influence of a cyanobacterial crude extract containing microcystin-LR on the physiology and antioxidative defence systems of different spinach variants. New Phytol..

[45] Corbel S., Mougin C., Martin-Laurent F., Crouzet O., Bru D., Nélieu S., Bouaïcha N. (2015). Evaluation of phytotoxicity and ecotoxicity potentials of a cyanobacterial extract containing microcystins under realistic environmental concentrations and in a soil-plant system. Chemosphere.

[46] Petrou M., Karas P.A., Vasileiadis S., Zafiriadis I., Papadimitriou T., Levizou E., Kormas K., Karpouzas D.G. (2020). Irrigation of radish (*Raphanus sativus* L.) with microcystin-enriched water holds low risk for plants and their associated rhizopheric and epiphytic microbiome. Environ. Pollut..

[47] Levizou E., Papadimitriou T., Papavasileiou E., Papadimitriou N., Kormas K.A. (2020). Root vegetables bioaccumulate microcystins-LR in a developmental stage-dependent manner under realistic exposure scenario: The case of carrot and radish. Agric. Water Manag..

[48] Lee S., Jiang X., Manubolu M., Riedl K., Ludsin S.A., Martin J.F., Lee J. (2017). Fresh produce and their soils accumulate cyanotoxins from irrigation water: Implications for public health and food security. Food Res. Int..

[49] Gu Y., Liang C. (2020). Responses of antioxidative enzymes and gene expression in *Oryza sativa* L. and *Cucumis sativus* L. seedlings to microcystins stress. Ecotoxicol. Environ. Saf..

[50] Zhu J., Ren X., Liu H., Liang C. (2018). Effect of irrigation with microcystins-contaminated water on growth and fruit quality of *Cucumis sativus* L. and the health risk. Agric. Water Manag..

[51] El Khalloufi F., Oufdou K., Lahrouni M., Faghire M., Peix A., Ramírez-Bahena M.H., Vasconcelos V., Oudra B. (2013). Physiological and antioxidant responses of *Medicago sativa*-rhizobia symbiosis to cyanobacterial toxins (Microcystins) exposure. Toxicon.

[52] Cao Q., Steinman A.D., Wan X., Xie L. (2018). Combined toxicity of microcystin-LR and copper on lettuce (*Lactuca sativa* L.). Chemosphere.

[53] Levizou E., Statiris G., Papadimitriou T., Laspidou C.S., Kormas K.A. (2017). Lettuce facing microcystins-rich irrigation water at different developmental stages: Effects on plant performance and microcystins bioaccumulation. Ecotoxicol. Environ. Saf..

[54] Cao Q., Steinman A.D., Wan X., Xie L. (2018). Bioaccumulation of microcystin congeners in soil-plant system and human health risk assessment: A field study from Lake Taihu region of China. Environ. Pollut..

[55] Lahrouni M., Oufdou K., Faghire M., Peix A., El Khalloufi F., Vasconcelos V., Oudra B. (2012). Cyanobacterial extracts containing microcystins affect the growth, nodulation process and nitrogen uptake of faba bean (*Vicia faba* L., Fabaceae). Ecotoxicology.

[56] Lahrouni M., Oufdou K., El Khalloufi F., Benidire L., Albert S., Göttfert M., Caviedes M.A., Rodriguez-Llorente I.D., Oudra B., Pajuelo E. (2016). Microcystin-tolerant Rhizobium protects plants and improves nitrogen assimilation in *Vicia faba* irrigated with microcystin-containing waters. Environ. Sci. Pollut. Res..

[57] Xiang L., Li Y.-W., Wang Z.-R., Liu B.-L., Zhao H.-M., Li H., Cai Q.-Y., Mo C.-H., Li Q.X. (2020). Bioaccumulation and Phytotoxicity and Human Health Risk from Microcystin-LR under Various Treatments: A Pot Study. Toxins.

[58] Chen J., Song L., Dai J., Gan N., Liu Z. (2004). Effects of microcystins on the growth and the activity of superoxide dismutase and peroxidase of rape (*Brassica napus* L.) and rice (*Oryza sativa* L.). Toxicon.

[59] Kuang X., Gu J.D., Tie B.Q., Yao B., Shao J. (2016). Interactive effects of cadmium and Microcystis aeruginosa (cyanobacterium) on the growth, antioxidative responses and accumulation of cadmium and microcystins in rice seedlings. Ecotoxicology.

[60] Zhu X., Shen Y., Chen X., Hu Y.O.O., Xiang H., Tao J., Ling Y. (2016). Biodegradation mechanism of microcystin-LR by a novel isolate of Rhizobium sp. TH and the evolutionary origin of the mlrA gene. Int. Biodeterior. Biodegrad..

[61] Ramani A., Rein K., Shetty K.G., Jayachandran K. (2012). Microbial degradation of microcystin in Florida’s freshwaters. Biodegradation.

[62] Chen W., Song L., Gan N., Li L. (2006). Sorption, degradation and mobility of microcystins in Chinese agriculture soils: Risk assessment for groundwater protection. Environ. Pollut..

[63] Cao Q., Steinman A.D., Yao L., Xie L. (2018). Effects of light, microorganisms, farming chemicals and water content on the degradation of microcystin-LR in agricultural soils. Ecotoxicol. Environ. Saf..

[64] Cao Q., Steinman A.D., Su X., Xie L. (2017). Effects of microcystins contamination on soil enzyme activities and microbial community in two typical lakeside soils. Environ. Pollut..

[65] Xiang L., Li Y.-W., Liu B.-L., Zhao H.-M., Li H., Cai Q.-Y., Mo C.-H., Wong M.-H., Li Q.X. (2019). High ecological and human health risks from microcystins in vegetable fields in southern China. Environ. Int..

[66] Wu X., Xiao B., Li R., Wang C., Huang J., Wang Z. (2011). Mechanisms and factors affecting sorption of microcystins onto natural sediments. Environ. Sci. Technol..

[67] Miller M.J., Critchley M.M., Hutson J., Fallowfield H.J. (2001). The adsorption of cyanobacterial hepatotoxins from water onto soil during batch experiments. Water Res..

[68] Mohamed Z.A., Al Shehri A.M. (2009). Microcystins in groundwater wells and their accumulation in vegetable plants irrigated with contaminated waters in Saudi Arabia. J. Hazard. Mater..

[69] Cevallos-Casals B.A., Cisneros-Zevallos L. (2010). Impact of germination on phenolic content and antioxidant activity of 13 edible seed species. Food Chem..

[70] Tarasevičienė Ž., Viršilė A., Danilčenko H., Duchovskis P., Paulauskienė A., Gajewski M. (2019). Effects of germination time on the antioxidant properties of edible seeds. CyTA J. Food.

[71] Pereira S., Saker M.L., Vale M., Vasconcelos V.M. (2009). Comparison of sensitivity of grasses (*Lolium perenne* L. and *Festuca rubra* L.) and lettuce (*Lactuca sativa* L.) exposed to water contaminated with microcystins. Bull. Environ. Contam. Toxicol..

